# Willingness to help women victims of intimate partner violence in a Spanish context: Differential factors, interactions and predictors

**DOI:** 10.1371/journal.pone.0307274

**Published:** 2024-07-18

**Authors:** Ainara Nardi-Rodríguez, Andrés Sánchez-Prada, Carmen Delgado-Álvarez, Esperanza Bosh-Fiol, Leila I. Vázquez-González, Victoria A. Ferrer-Pérez

**Affiliations:** 1 Behavioural and Health Sciences Department, Miguel Hernández University, Elche, Spain; 2 Psychology Department, Pontifical University of Salamanca, Salamanca, Spain; 3 Psychology Department, University of Balearic Island, Palma de Mallorca, Spain; Ariel University, ISRAEL

## Abstract

This article presents two cross-sectional studies that group the most relevant (and potential) factors contemplated in the bystander literature on Intimate Partner Violence Against Women, (IPVAW). We analyzed their relationship with the intention to respond to hypothetical scenarios with specific helping behaviors based on the witnesses’ gender, political ideology and on the bystander effect (study 1). We also studied them as predictors of helping behaviors (study 2). In total, 1,563 Spanish people participated in study 1 and 755 Spanish people in study 2. Participants had to study an IPVAW vignette (with a single bystander or multiple bystanders) and a control scenario (a robbery with a woman as victim or a man) and assess the perceived severity of the situation, the perceived responsibility of the victim and the aggressor(s), the personal perceived responsibility of the bystander and the intention to perform 8 helping behaviors. They also fulfilled a social desirability scale (study 1 and 2), the Inventory of Distorted Thoughts about Women and Violence and the Scale on Gender Ideology (study 2). Women tend to assess the IPVAW scenario in a way that favors displaying active helping behaviors to a greater extent than men. An individual’s political opinion has also shown to affect the assessment and, to a lesser extent, the intention to help an IPVAW victim. The bystander effect only takes place when negative attitudes are present. When analyzing the interaction between the type of violence (gender versus non-gender-based violence) and the above-mentioned variables, the results tend to confirm previous studies. Regarding the predictors of the helping behaviors, perceived personal responsibility is key, together with victim blaming attitudes or the perceived severity of the situation. This study expands the knowledge on bystander behaviors in IPVAW contexts and offers elements to work on awareness campaigns.

## Introduction

Intimate partner violence against women (IPVAW) is a public health issue of epidemic proportions [1]. It affects at least 30% of women worldwide [2] and approximately 20% of the women living in Europe [3]. In Spain, according to the last macro-study, 32.4% of the female population above 15 years of age has suffered psychological, physical or sexual violence from a partner [4]. This social and health issue has devastating consequences for women, children and society in general [5], imposing an annual cost of nearly 152 billion euros for Europe [6].

To take steps towards the eradication of IPVAW, promoting helping behaviors among the community can be an effective strategy [7, 8]. Living in a community that intervenes when their members witness IPVAW has a twofold protective factor towards victims: it stops the violent incident at that moment; and exerts social pressure on the aggressors, reducing their likelihood to use violence [9]. Hence, the presence of passive non-helping behaviors among citizens, such as ignoring the event, need to be replaced by active helping behaviors, such as aiding the victim or reporting the aggression. According to a recent empirical ex–post facto study analyzing secondary data pulled from sociological studies performed in Spain between 2005 and 2020, about 30% of the general and younger population are aware of a friend or neighbor who suffers IPVAW; however, the performance of active bystander responses, such as reporting the situation to the police, were very low [10]. In 2022, not a single call made to the main Spanish hotline for IPVAW support and emergencies (N = 102.391) was made by unknown persons and only 2.3% by other persons different from the victim’s family or inner circle [4]. Although the Spanish public’ s intention to help has risen up to 90% [10], the aforementioned data highlight that in real situations, helping behaviors are not so frequent and thus need to be boosted. It is possible that personal and contextual factors in such situations influence intentions by boosting or impeding the manifestation of active bystander behaviors.

According to the Bystander Intervention Model [11] helping behaviors are a result of a 5-step cognitive process: notice the event; interpret it as an issue; assume personal responsibility to intervene as a witness; know how to help; and finally, translate such willingness into real helping behavior. The authors of the model focus on the ‘bystander effect’ as a contextual barrier for displaying helping behaviors. It is supposed to take place when a witness’s responsibility to act diffuses because of the presence of more witnesses. Burn proposed an explanatory model of helping behaviors in contexts of violence against women [12], specifically in sexual assault situations, which included variables such as the witness’s gender, the bystander effect, the attribution of worthiness (related to victim blaming), not knowing the victim or aggressor or being in a crowded social situation, such as a party, which can impede a bystander from noticing the event. Banyard presented a wider framework that integrated the ecological models to the explanation of helping behaviors in sexual violence situations [13]. According to this model, at an individual level, cognitions and attitudes such as personal beliefs on rape myths can affect helping behaviors. Other cognitions that may influence, are bystander’s perception of their own responsibility to act, and the degree of victim and perpetrator blaming. Contextual factors such as the severity of the situation, knowing or not the victims or self-identification processes with the victims can impact at an individual level by activating emotions, which can also trigger helping behaviors. The person’s gender, her/his cost-benefit assessment of intervening and his/her perceived self-efficacy for intervening can also impact on helping behaviors.

At a microsystem level, Banyard’s approach contemplates the influence of family and peers on helping behaviors through different mechanisms [13]. For instance, when a person’s inner context does not support them intervening, then the “audience inhibition effect” or fear of embarrassment can take place, hindering the person from acting. Also, an “evaluation apprehension” effect can occur when bystanders either perceive that others can assess them negatively for intervening because they considered it unnecessary or they disagreed with the bystander’s choice of strategy. Another important variable at this level is the characteristics of the situation itself; for instance, the ability or difficulty in perceiving a critical situation (i.e. crowded or noisy places), whether the bystander knows the victim and/or the perpetrator, the number of bystanders or the location where the aggression occurs (settings can elicit different behaviors). Finally, at an exosystem and macrosystem level, community attitudes, the behavior of community leaders, or the presence of rape centers can also boost or hinder helping behaviors. According to Banyard’s model, in line with ecological approaches, all levels interact and are interdependent. Thus, within time, changes in one level affect the others. Although wide approaches are needed to eradicate violence against women, in IPVAW contexts, the role of intrapersonal and situational variables [14–16] are still studied since they need further clarification.

At a personal level, some studies point to the gender of the witnesses as a factor that affects helping behaviors, showing that women tend to intervene in greater measure than men or report a greater willingness to do so [17–19]. In contrast, others have found that men intervene in greater measure than women in high-risk situations [20, 21] or even that gender is not a determining factor [22]. Thus, the role of the witnesses’ gender remains unclear.

Continuing with the personal level, perceiving the situation as severe has also been considered a pre-conditional factor to display active helping behaviors [11, 18, 23]. This factor, compared to others, has received less attention by European scholars [24]. In comparison to other types of violence, people perceive less urgency in acting in IPVAW contexts [15] since they are not so often perceived as emergencies [25] because of people’s attitudes towards the issue, another factor behind (non-) helping behaviors [23]. A large European meta-analysis of 40 studies from 19 different countries pointed out that victim-blaming or sexist attitudes were still prevalent among citizens [26]. Holding victims responsible for the violent event (victim-blaming attitudes) is an attitudinal variable that influences the intention of a bystander to intervene. In a recent Australian study, the perceived responsibility of both the victims and perpetrators accounted for the unique variance in willingness to intervene [25]. Many studies corroborate that victim-blaming attitudes impede active helping behaviors from taking place, inhibit victims from searching for help, and reinforce the behavior of perpetrators [22, 27, 28]. In contrast, when the aggressors are perceived as responsible for the violent situations, witnesses are more prone to help victims [16, 25]. According to a study conducted in Spain [23], the population that considered IPVAW to be a private matter scored higher in victim-blaming attitudes, held more hostile sexist beliefs and were less prone to help. In addition to the perceived responsibility of the victims and aggressor(s), personal responsibility to act is also considered a central personal factor that affects helping behaviors [18]. It is a required step to help a victim [11] and has been related to the perceived severity of violence [24]. In other words, the greater the perceived severity, the greater the chances for feeling responsible to act. However, as stated above, people feel less urgency in intervening in IPVAW contexts [15, 25] and perhaps lower perceived responsibility to intervene.

Pavlou & Knowles [29] also found that people who intend to maintain and defend traditional gender roles present greater victim blaming attitudes, lower perceived responsibility of aggressors and higher levels of justification of IPVAW. Thus, gender ideology could influence helping behavior. In this same line, a Spanish study found that conservative people with right-wing political tendencies presented lower levels of rejection of IPVAW [30]. Considering that gender biases and discrimination processes in decision making situations related to circumstances other than IPVAW have been found (i.e., in healthcare contexts; see [31]), and considering all the above-mentioned negative attitudes specific to IPVAW that people can hold (i.e. victim-blaming attitudes, sexism or perceived severity), it could be that people with right wing political ideologies are less prone to intervene in these situations than in non-gender based situations, such as robberies. Conversely, other studies have discarded political ideology as a predictor of formally reporting an IPVAW situation (i.e., [16]). In the reviewed literature, studies analyzing the relationship between political ideology and bystander behaviors in IPVAW contexts are still scarce, especially in Spain. Also, no studies have been found with regard to the extent to which the type of situation (gender vs non-gendered) interacts with the gender, political opinion and the bystander effect of the witness.

Finally, at a contextual level, the “bystander effect” has been considered a well-established factor behind (non-)helping behaviors [32]. Such an effect refers to the fact that the more witnesses are present the less likely it is that they will intervene [33]. However, several studies have shown that the activation of this effect is also subjected to the perceived severity of the situation [18, 24, 33, 34]. In the few laboratory-settings studies that reproduced fictitious dangerous situations, the bystander effect did not occur [35]. Similar results were found in a meta-analysis conducted by Fischer et al [36].

### The present study

As we have argued, some variables seem to play a key role in the activation of helping behaviors in IPVAW cases. In general terms, studies have analyzed a limited number of variables related to bystander behaviors and in some cases, results are inconclusive [16], requiring further clarification of their role. To this end, we performed two cross-sectional studies grouping the most relevant factors contemplated in the bystander literature for IPVAW contexts (as well as potentially relevant factors) to analyze them with regard to specific active bystander behaviors (helping behaviors) and negative or passive bystander behaviors (non-helping) in a Spanish context. The first study, aimed to analyze the relationship between the aforementioned variables in an IPVAW hypothetical scenario. More specifically: (1) Analyze, according to the witnesses’ gender, their assessment of the situation (the perceived severity and responsibility of the aggressor, the victim and the bystander) and their intention to respond to IPVAW; (2) Study, according to political opinion, the assessment and intention to respond to IPVAW; (3) Analyze the bystander effect (number of bystanders) on the assessment and intention to respond to IPVAW; (4) Analyze the effect of the type of violence (gender-based or not) on the assessment and intention to respond to IPVAW, and; (5) Study the effect of the interaction between the type of violence (gender-based or not) and other variables (bystander’ gender, bystander political opinion, and number of bystanders). The purpose of the second study was to explore the predictors of bystander intentions to respond in an IPVAW hypothetical scenario. Studies in this area have mainly examined the factors that can act as barriers and facilitators of intervening in IPVAW situations (i.e. [37, 38]) but few have focused on identifying predictors of bystander behaviors in IPVAW scenarios [25, 38–40], including one in Spain [41]. However, in general terms, these studies did not analyze the predictors of diverse specific (non-) helping behaviors. Focusing on specific behaviors increases the precision with which they can be predicted [42]. Therefore, while the first study focused on analyzing how different individual and contextual factors (such as gender, political ideology, or bystander effect) can influence bystanders’ responses, the second one aims to identify whether some relevant variables studied in previous research on bystander behavior (such as sexist attitudes or gender ideology) are predictors of different bystander responses.

## Study 1. Factors related to helping behaviors in IPVAW contexts

### Method

#### Participants

An opportunity sample of 1,563 Spanish people with an average age of 33.38 years (*SD* = 14.69; range: 18–77) took part in this study, of which 20.6% (*n* = 322) were men and 79.4% women (*n* = 1.241). The majority had university studies (53%) followed by secondary studies (29.8%). Regarding their political opinion, 59.6% considered themselves left-wing, 15.1% center-wing, 9.3% right-wing, and 16.0% add some other options.

#### Procedure

The research protocol for this study was approved by the Bioethics Committee of the University of the Balearic Islands (Ref. 123CER19, 19^th^ November 2019). Participation was voluntary and anonymous, and no incentives were offered to the participants. The only prerequisites to participate were being of age and understanding the language. Upon initiating the survey, a text appeared explaining the aims and conditions of the study. To access to the questionnaire answer sheet, participants had to click a checkbox and explicitly agree to participate in the study. If not, participants were unable to answer the questionnaire and their participation was terminated. Given that the proposed methodology consisted of the compilation of responses to a series of anonymous questionnaires through an online application and that participants would not be asked for personal data or any information that could reveal their identification, the Committee considered it unnecessary to sign a consent report. The recruitment period for this study started on February 24, 2022 and ended on May 17, 2022.

A non-probability convenience sample was used. The questionnaire used was designed on the Lime Survey platform and disseminated through social network sites used by the researchers and their collaborators. Participants were provided with a link to the webpage where the questionnaire could be found and the survey was optimized for tablet and smartphone respondents. An introductory text about the objectives and conditions of the study was included and access to the answer sheet implied prior agreement of the participants to take part in the study. Lime Survey randomly assigned participants to both scenarios (robbery and IPVAW) with one witness (*n* = 806 participants) or several witnesses (*n* = 752 participants). Each survey took approximately 30 to 60 min to complete. It is also important to note that the case scenarios were presented in random order for each participant (using the randomization feature used in the Lime Survey platform).

The response rate was 66.04% (1,577 people completed the questionnaires from 2,388 that initiated them), a high rate compared with the average obtained by Wu, Zhao and Fils-Aime [43]. Among the respondents, 0.89% of the respondents (14 people) identified as outside of the male/female gender binary. Due to this insufficient sample size, they were not included in this study.

#### Instruments

The following questionnaires were used:

*Brief questionnaire with sociodemographic questions*. Participants were asked for their age, gender (self-categorized by participants in an open-ended item), and political ideology (participants could choose between three response options, right-wing, center or left-wing position, or add their own option to the list).

*Social desirability scale*. Given the effect of social desirability in self-reported measures of IPVAW [12], we used the reduced version of the SDS [44], the M-CSDS-10 version [45], adapted to a Spanish context by Lila et al [46]. It is composed of 10 items with a dichotomous response scale (true/false). Its score ranges from 0 to 10 points (α = .63).

*The Questionnaire of Intention to Help in VAW Cases (QIHVC)*. The psychometric properties of the instrument, designed in a Spanish context, are described in a previous study (see [47]). The questionnaire was submitted to a Delphi study with experts in violence against women and tested with a sample of Spanish university students. It presents a good content validity and seems an adequate and sensible tool to capture differences in participants answers when they are in front of a common form of violence or in front of an IPVAW case. It includes the description of different hypothetical scenarios of gender-based violence (including IPVAW) and a common form of violence (robbery situation). It should be noted that the robbery scenario reflected in fact a case of theft, but we decided not to use this highly technical term because the Spanish population usually refers to this type of situations as robbery. In order to assess the bystander effect under different circumstances, some participants had to respond to the questionnaire where they were the only witness (n = 806) while others had to respond in situations where they were accompanied by more witnesses (n = 752). In both cases they were asked about the following points: the perceived severity of the situation (on a 7-point scale from *Not severe at all* to *Very Severe*); the victim’s perceived responsibility; the aggressor’s responsibility, and; the participant’s responsibility to intervene as bystander (on a 7-point scale from *Not responsible at all* to *Completely responsible*). They were also asked how likely they thought they were to perform each of the 4 active-positive and 4 negative or passive bystander responses studied if they were to witness this situation (on a 7-point scale, from *Not probable* to *Highly probable*). The active bystander behaviors were: *Confront the perpetrator*; *call the police/notify to the authorities*; *help the victim*, *and; ask other people for help*. In contrast, the negative or passive bystander behaviors were: *to reproach the victim* for her/his actions; *not know what to do*; *get blocked*; *do nothing because it’s not my business*, and; *do nothing out of fear*.

#### Data analysis

With regard to the first two objectives and their analysis based on participants’ gender, the Student’s t-test was applied for independent samples, previously checking the homoscedasticity of the variables, and correcting the degrees of freedom when the variances were not homogeneous. On the other hand, the comparison by political opinion was made using an ANOVA of one factor. For post-hoc comparisons, the Scheffe test was used for variables with homogeneous variances due to its greater robustness in samples of different sizes, while the Games-Howell test was used for variables without homoscedasticity. Regarding the third objective, the bystander effect was analyzed using ANCOVA, and regarding the fourth aim, comparing the answers to the IPVAW scenario with common violence (robbery), a Repeated Measures Model was used. In both cases, the effect of social desirability was controlled, considering that the presence of other people and social awareness campaigns on IPVAW could affect the responses in the sense of "socially desirable". The interaction between the type of violence and other variables (three intersubject variables: two personal-gender and political opinion-, and one situational -the number of bystanders-) were analyzed using a Mixed Repeated Measures Model for each of the three types of variables. As a measure of effect size, we used eta square. Considered, as usual, that η^2^ = 0.01 indicates a small effect, η^2^ = 0.06 a medium effect and η^2^ = 0.14 a large effect [48]. Data analysis was performed using the SPSS v. 25.

### Results

#### Analysis by gender

The results of the comparison of the assessment and intention to respond to a case of IPVAW according to gender are shown in Table 1.

**Table 1:** Analysis by gender.

Women (n = 1,239)		Mean *(SD)*	Lévène	t	*p*	η^2^
Men (n = 319)			*(p)* *	Student		
IPVAW scenario assessment						
Perceived Severity	Women	6.54 *(0*.*79)*	**.000**	6.926	**.000**	.046
	Men	6.06 *(1*.*17)*				
Victim’s Responsibility	Women	1.49 *(1*.*01)*	**.000**	-3.740	**.000**	.011
	Men	1.76 *(1*.*17)*				
Aggressor’s Responsibility	Women	6.78 *(0*.*74)*	**.000**	3.574	**.000**	.009
	Men	6.60 *(0*.*84)*				
Bystander’s Responsibility	Women	5.38 *(1*.*52)*	.789	4.079	**.000**	.011
	Men	4.99 (1.60)				
Bystander behaviors in an IPVAW scenario						
Reproach the victim	Women	1.42 *(1*.*20)*	.471	0.154	.878	---
	Men	1.40 *(1*.*03)*				
Confront perpetrator	Women	4.15 *(2*.*00)*	.329	-2.785	**.005**	.005
	Men	4.50 *(1*,*91)*				
Call the police	Women	6.06 *(1*,*42)*	**.000**	4.478	**.000**	.015
	Men	5.60 *(1*.*67)*				
Help the victim	Women	6.45 *(1*.*09)*	**.000**	5.330	**.000**	.022
	Men	6.03 *(1*.*30)*				
Ask for help	Women	5.91 *(1*,*57)*	**.000**	6.875	**.000**	.037
	Men	5.10 *(1*.*93)*				
Get blocked	Women	2.42 *(1*.*59)*	.483	1,973	**.049**	.002
	Men	2.23 *(1*.*55)*				
It’s not my business	Women	1.53 *(1*.*07)*	**.012**	-2.604	**.009**	.005
	Men	1.71 *(1*.*14)*				
Nothing out for fear	Women	2.17 *(1*.*48)*	**.000**	4.870	**.000**	.012
	Men	1.77 *(1*.*24)*				

* If p < .05 in Levene’s test,

Student’s t test is taken, not assuming equal variances (correction of degrees of freedom).

In all the variables analyzed, significant differences were obtained, except for the intention to reproach the victim. Regarding the assessment of the situation, the women considered the scene described as more serious, attributed more responsibility to the aggressor as the cause of IPVAW, and perceived themselves as more responsible to intervene. Men, on the other hand, attributed more responsibility to the victim. Regarding the intention to intervene in an IPVAW case, women scored significantly higher in helping responses (*call the police/notify to the authorities*, *help the victim*, and *ask other people for help*), and paralysis (*not know what to do*, *get blocked*, and *do nothing out of fear*), while men scored higher in confrontational responses (*confront the aggressor*) and disagreement (*do nothing because it’s not my business*).

#### Analysis according to political ideology

To compare the responses by political opinion, the responses of people who declared themselves to be left, center, and right-winged were analyzed. Those who gave other responses were excluded due to the small sample size (*n* = 49). The results obtained are shown in Table 2.

**Table 2:** Analysis by political opinion.

Left (n = 930)		Mean *(SD)*	Lévène (p)*	*F* (2,1307)	*p*	η^2^
Center (n = 235)						
Right (n = 145)						
IPVAW scenario assessment						
Perceived Severity	Left	6,51 *(0*.*84)*	**.000**	6.603	**.001**	.010
	Center	6,38 *(0*.*86*)				
	Right	6,26 *(1*.*08)*				
Victim’s Responsibility	Left	1.40 *(0*.*87)*	**.000**	17.935	**.000**	.027
	Center	1.66 *(1*.*11)*				
	Right	1.86 *(1*.*32)*				
Aggressor’s Responsibility	Left	6.84 *(0*.*57)*	**.000**	8.162	**.000**	.012
	Center	6.77 *(0*,*66)*				
	Right	6.62 *(0*,*86)*				
Bystander’s Responsibility	Left	5.49 *(1*.*47)*	.616	12.034	**.000**	.018
	Center	4.97 *(1*.*55)*				
	Right	5.20 *(1*.*57)*				
Bystander behaviors in an IPVAW scenario						
Reproach the victim	Right	1.28 *(0*.*96)*	**.000**	6.379	**.002**	.010
	Center	1.45 *(1*.*20)*				
	Left	1.57 *(1*.*25)*				
Confront perpetrator	Right	4.14 *(1*.*95)*	.732	5.633	**.004**	.009
	Center	4.40 *(2*.*01)*				
	Left	4.68 *(1*.*94)*				
Call the police	Right	6.06 *(1*.*41)*	**.000**	3.581	**.028**	.005
	Center	5.78 *(1*.*62)*				
	Left	5.93 *(1*.*46)*				
Help the victim	Right	6.42 *(1*.*09)*	.741	0.388	.679	---
	Center	6.41 *(1*.*05)*				
	Left	6.33 *(1*.*08)*				
Ask for help	Right	5.84 *(1*.*60)*	**.000**	2.884	.056	---
	Center	5.66 *(1*.*80)*				
	Left	5.52 *(1*.*85)*				
Get blocked	Right	2.49 *(1*.*59)*	**.042**	5.678	**.004**	.009
	Center	2.25 *(1*.*56)*				
	Left	2.07 *(1*.*43)*				
It’s not my business	Right	1.46 *(0*.*98)*	**.000**	8.043	**.000**	.012
	Center	1.69 *(1*.*24)*				
	Left	1.76 *(1*.*17)*				
Nothing out for fear	Right	2.12 *(1*.*42)*	.303	2,431	.088	---
	Center	2.04 *(1*.*46)*				
	Left	1.85 *(1*.*29)*				

* For post-hoc comparisons, the Scheffe test is performed if p>.05 in the Levene test and the Games-Howell test is performed if p ≤ .05.

Statistically significant differences were obtained in the four variables related to the IPVAW assessment. Post-hoc tests showed that, compared to left-wing people, those identifying as right-wing attributed less seriousness to IPVAW (p = .004), greater responsibility to the victim (p < .001), less responsibility to the aggressor as the cause of IPVAW (p < .001), and less responsibility to bystanders in intervening (p = .004). It should be noted that people who consider themselves to hold a centrist ideological opinion do not differ from the other two political options in terms of their assessment of the seriousness of the IPVAW situation or the responsibility to intervene attributed to the bystanders (p > .05). Instead, they form a homogeneous group with the people with right-wing ideology, attributing more responsibility to the victim (p = .001) and less responsibility to the aggressor as the cause of IPVAW (p = .045) than the people with left-wing ideology.

Regarding the intention to intervene in an IPVAW case, there are no significant differences (p > .05) in the responses *to help the victim*, *ask for help*, or *do nothing out of fear*. Post-hoc comparisons for the answers in which there are significant differences show that right-wing people would be more likely to choose answers such as *reproach the victim* (p = .024), *confront the aggressor* (p = .009), or *do nothing because it’s not my business* (p = .012) and, to a lesser extent, a response such as *do nothing out of fear* (p = .004). People located in the center of the ideological spectrum obtained intermediate scores that do not differ significantly from people on the right or on the left side of the spectrum for all responses (p >.05), except for *calling the police*, which they chose significantly less than left-wing people (p = .044).

#### The bystander effect

To analyze the bystander effect, we compared the responses to both IPVAW conditions: the IPVAW scenario with only one witness (the participant) and with more than one witness (the participant and other bystanders). The results obtained are shown in Table 3.

**Table 3:** The bystander effect.

Only one (n = 806)		Mean *(SD)*	Lévène *(p)*	Covariate *(p)*	*F* (2,1555)	*p*	η^2^
> than one (n = 752)							
IPVAW scenario assessment							
Perceived Severity	Only 1	6.53 *(0*.*83)*	**.000**	.074	16.613	**.000**	.011
	>than 1	6.34 *(0*.*97)*					
Victim’s Responsibility	Only 1	1.52 *(1*.*09)*	.949	.155	1.016	.314	---
	>than 1	1.57 *(1*.*00)*					
Aggressor’s Responsibility	Only 1	6.78 *(0*.*72)*	**.002**	.386	3.508	.061	---
	>than 1	6.71 *(0*.*80)*					
Bystander’s Responsibility	Only 1	5.45 *(1*.*52)*	.787	**.007**	14.809	**.000**	.009
	>than 1	5.14 *(1*.*54)*					
Bystander behaviors in an IPVAW scenario							
Reproach the victim	Only 1	1.37 *(1*.*17)*	**.048**	.568	2.316	.128	---
	>than 1	1.46 *(1*.*15)*					
Confront perpetrator	Only 1	4.28 *(2*.*00)*	.448	**.007**	1.514	.219	---
	>than 1	4.15 *(1*.*97)*					
Call the police	Only 1	6.01 *(1*.*47)*	.214	**.003**	1.891	.169	---
	>than 1	5.91 *(1*.*51)*					
Help the victim	Only 1	6.42 *(1*.*11)*	**.029**	**.001**	3.470	.063	---
	>than 1	6.31 *(1*.*18)*					
Ask for help	Only 1	5.78 *(1*.*72)*	.263	.157	0.726	.394	---
	>than 1	5.70 *(1*.*64)*					
Get blocked	Only 1	2.33 *(1*.*60)*	.975	**.000**	1.730	.189	---
	>than 1	2.44 *(1*.*56)*					
It’s not my business	Only 1	1.48 *(1*.*01)*	**.000**	**.002**	9.552	**.002**	.006
	>than 1	1.65 *(1*.*15)*					
Nothing out for fear	Only 1	2.02 *(1*.*42)*	.169	**.000**	3.397	.066	---

Regarding the assessment of the IPVAW scenario, it is observed that, when controlling for the effect of social desirability, those who have responded to the single witness condition perceive the IPVAW situation as more serious and attribute a greater responsibility of intervention to the bystander; that is, to themselves. With regard to the intention of responding to a case of IPVAW, significant differences were only obtained for the response *do nothing because it’s not my business* in the multiple bystander scenario.

#### Effect of the type of violence

To analyze the effect of the type of violence, the responses to the QIHVC were compared in relation to a common form of violence (a robbery where the victim was a woman) and the IPVAW as gender-based violence. The results obtained are shown in Table 4.

**Table 4:** Effect of the type of violence.

	(n = 1558)	Mean *(SD)*	Covariate *(p)*	*F* (1,1555)	*p*	η^2^
Scenarios’ assessment						
Perceived Severity	Robbery	5.47 *(1*.*39)*	.180	650.07	**.000**	.051
	IPVAW	6.44 *(0*.*90)*				
Victim’s Responsibility	Robbery	2.94 *(1*.*76)*	.508	71.62	**.000**	.044
	IPVAW	1.55 *(1*.*05)*				
Aggressor’s Responsibility	Robbery	6.84 *(0*.*64)*	.999	2.08	.150	---
	IPVAW	6.75 *(0*.*76)*				
Bystander’s Responsibility	Robbery	4.73 *(1*.*67)*	**.016**	38.33	**.000**	.024
	IPVAW	5.30 *(1*.*54)*				
Bystander behaviors in response to each violent situation						
Reproach the victim	Robbery	2.03 *(1*.*45)*	.478	26.63	**.000**	.017
	IPVAW	1.41 *(1*.*16)*				
Confront perpetrator	Robbery	4.34 *(1*.*94)*	.430	0.04	.951	---
	IPVAW	4.22 *(1*.*28)*				
Call the police	Robbery	5.78 *(1*.*96)*	.791	0.99	.321	---
	IPVAW	5.96 *(1*.*49)*				
Help the victim	Robbery	6.34 *(1*.*16)*	.359	1.44	.231	---
	IPVAW	6.37 *(1*.*15)*				
Ask for help	Robbery	5.75 *(1*.*60)*	.379	0.60	.438	---
	IPVAW	5.74 *(1*.*68)*				
Get blocked	Robbery	2.42 *(1*.*59)*	.930	0.12	.735	---
	IPVAW	2.38 *(1*.*58)*				
It’s not my business	Robbery	1.67 *(1*.*19)*	**.037**	8.72	**.003**	.006
	IPVAW	1.57 *(1*.*09)*				
Nothing out for fear	Robbery	2.05 *(1*.*42)*	.547	0.95	.330	---
	IPVAW	2.09 *(1*.*44)*				

Regarding the assessment of the IPVAW situation, it is observed that when controlling for the effect of social desirability, IPVAW is considered as significantly more serious than the case of robbery. In addition, the victim in the IPVAW scenario is perceived as less responsible and a greater responsibility is attributed to the bystander to intervene. In terms of response intention, significant differences were only obtained for *reproach the victim* and *do nothing because it’s not my business*, which are less likely in the IPVAW situation than in the robbery scenario.

#### Interaction between type of violence and other variables

The results obtained by analyzing the interaction between the type of violence (IPVAW or common violence) and the variables studied are shown in Table 5.

**Table 5:** Interaction between type of violence and other variables.

	Violence*Gender	Violence*Political	Violence*Bystanders
	Interaction	Interaction	Interaction
	*F* (1,1555)	*F* (1,1555)	*F* (1,1555)
Violence assessment			
Perceived Severity	*F* = 14.080	*F* = 9.291	*F* = 0.312
	*p* = **.000**	*p* = **.000**	*p* = .576
	[η^2^= .009]	[η^2^= .014]	
Victim’s Responsibility	*F* = 0.574	*F* = 1.138	*F* = 0.088
	*p* = .449	*p* = .321	*p* = .766
Aggressor’s Responsibility	*F* = 3.102	*F* = 3.349	*F* = 0.234
	*p* = .078	*p* = **.035**	*p* = .629
		[η^2^= .005]	
Bystander’s Responsibility	*F* = 8.537	*F* = 12.172	*F* = 0.916
	*p* = .**004**	*p* = **.000**	*p* = .339
	[η^2^= .005]	[η^2^= .018]	
Helping behaviors			
Reproach the victim	*F* = 4.452	*F* = 3.736	*F* = 0.051
	*p* = **.035**	*p* = **.024**	*p* = .822
	[η^2^= .003]	[η^2^= .006]	
Confront perpetrator	*F* = 6.510	*F* = 0.351	*F* = 0.579
	*p* = **.011**	*p* = .704	*p* = .447
	[η^2^= .004]		
Call the police	*F* = 4.197	*F* = 3.900	*F* = 1.438
	*p* = **.041**	*p* = **.020**	*p* = .231
	[η^2^= .003]	[η^2^= .006]	
Help the victim	*F* = 9.393	*F* = 1.169	*F* = 0.633
	*p* = **.002**	*p* = .189	*p* = .426
	[η^2^= .006]		
Ask for help	*F* = 3.356	*F* = 1.504	*F* = 3.684
	*p* = .067	*p* = .349	*p* = .055
Get blocked	*F* = 0.834	*F* = 0.343	*F* = 0.489
	*p* = .361	*p* = .710	*p* = .485
It’s not my business	*F* = 1.729	*F* = 1.231	*F* = 0.509
	*p* = .189	*p* = .292	*p* = .476
Nothing out for fear	*F* = 1.378	*F* = 0.126	*F* = 3.673
	*p* = .241	*p* = .882	*p* = .055

The absence of interaction between the number of bystanders and the type of violence indicates that the number of bystanders equally affects the different aspects of the assessment of both violent scenarios and the intention to respond to them. On the other hand, the individual variables studied (gender and political opinion) do affect differently some aspects of the evaluation and behavioral intention, depending on the type of violence.

**Fig 1:**
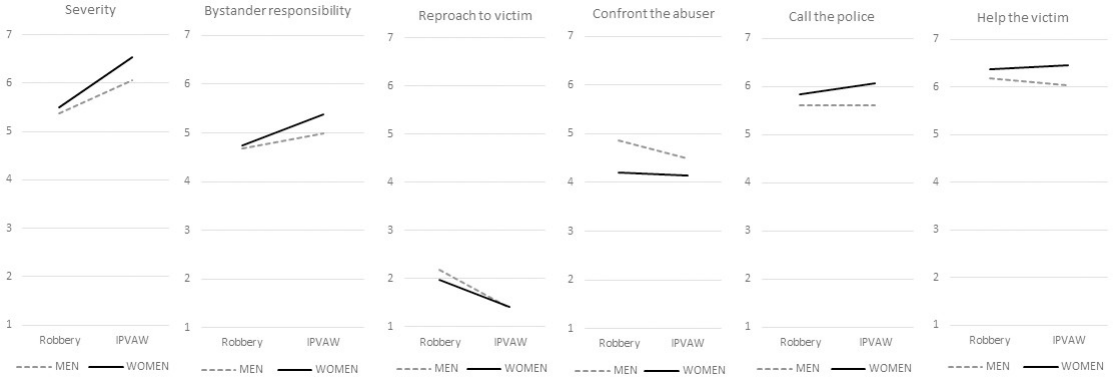
Interaction between type of violence and gender.

As can be seen (Fig 1), with regard to the perceived severity and bystander’s responsibility, a similar pattern is observed: there are no significant differences between men and women in the case of robbery, whereas an increased difference is observed in these scores for the IPVAW scenario, which are higher among women. With regard to response intention, two different patterns are observed: on the one hand, the intention to *reproach the victim* and *confront the aggressor* is greater among men for both types of violence, although it decreases in the case of IPVAW (especially for the response reproach the victim where men and women’ s scores are equal). Regarding *call the police/notify to the authorities* or *help the victim*, the intention is higher among women for both types of violence and even increases in the case of IPVAW (while for men it remains the same or even decreases when it comes to helping the victim).

**Fig 2:**
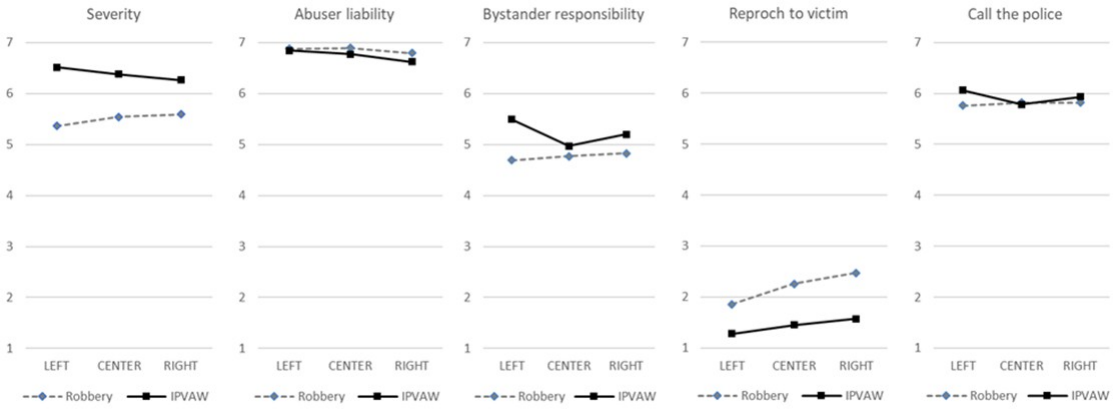
Interaction between type of violence and political opinion.

Regarding the interaction with political opinion (Fig 2), the perceived seriousness of IPVAW is higher than that of common violence in all cases, although this difference is reduced among people with center-wing ideology and, especially, of right-wing ideology. The responsibility of the aggressor is the same for both types of violence among people with left-wing ideas, while for people with center-wing ideas and, especially, for those with right-wing ideas, this responsibility lessens when it comes to IPVAW. The responsibility of the bystander to intervene is the same for both types of violence between people with center-wing ideas, while for people with right-wing ideas and, especially, for those with left-wing ideas, this responsibility is greater when it comes to IPVAW. Regarding the interaction between political ideology and the intention to respond, the intention to *reproach the victim* is lower in IPVAW cases for all people of any political opinion, but the difference with common violence (robbery) is greater between people from the center and from the right, who show more intention to answer in this sense than people of left-wing ideas. Finally, the intention to *call the police/notify to the authorities* is only higher in cases of IPVAW among people with left-wing ideas.

### Discussion

In line with previous studies, women showed a major tendency to display helping responses, especially non-confrontational active helping behaviors in contrast to men, who stood out in the confrontational response (i.e. *see* [17, 19, 23, 24, 49]. Thus, our results confirm such a tendency in women and men. In addition, women perceived the IPVAW scenario as more severe, perceived higher personal responsibility to act, and perceived victims as less responsible for the situation in contrast to aggressors who were held more responsible for the violence. As found in a recent review of European studies [24], women generally perceive IPVAW as a more serious issue than men, and are more aware of it (see [50]) which could explain why they hold victims less responsible for the violence, feel more responsible for intervening, and show a greater intention to actively help the victims.

This study also widens knowledge on how these variables change in a female or male bystander according to the type of violence that is being witnessed. While there are no differences by gender in the perceived severity and bystanders’ responsibility to act in the robbery scenario, women scored higher in the IPVAW scenario. Once again, this could be due to the fact that women tend to conceive of IPVAW as a more severe issue than men [24]. Also, the activation of group membership processes with victims could explain why women perceived a greater responsibility to act, as suggested by other authors [51]. In contrast, men were more likely to perceive IPVAW as a private matter, as found in previous studies [26]. These negative attitudes have been associated with negative or passive non-helping behaviors [23], which could explain why men showed a lower willingness to intervene compared to women.

Regarding bystander behaviors, the intention to display active helping behaviors is again higher in women than men, regardless of the type of violence, and increases in the case of IPVAW, which again could be related to the fact that women are more aware of the problem or because they feel they belong to the same group (women) as stated before [50, 51]. Whereas for men, confronting responses decreases in the case of the IPVAW scenario compared to the robbery scenario, which could be because they perceive IPVAW as less dangerous and thus display other helping active behaviors. *Reproaching the victim* was another negative or passive non-helping behavior that decreased in men when faced with the IPVAW scenario, which could reflect that they hold fewer victim blaming attitudes in these cases. Some studies point out that young and middle-aged adults (similar in age to our sample) hold fewer IPVAW supportive attitudes than adolescents and older people [10].

Regarding political ideology, although all tendencies believe IPVAW is a more severe issue than robbery, right-wing and center-wing people perceive less difference in the severity of the situations. Right-wing people, and especially left-wing people, perceive a higher responsibility to act in IPVAW situations compared to robbery, although no differences have been found on helping behaviors, except for *reproaching the victim*, which would be greater between people from the center and those from the right. Focusing exclusively on the IPVAW scenario, right-wing people perceive IPVAW as less serious and feel less responsible to intervene than left wing people. In addition, together with center-wing people, they perceive victims as more responsible for the situation and aggressors less responsible for it. The latter perception is accentuated in IPVAW situations compared to gender neutral situations. Also, right wing people would, in greater measure than left wing people, *reproach the victims* or *do nothing because it is not their concern*. All the above-mentioned attitudes and behaviors are coherent with conservative ideology, which advocates maintaining traditional gender roles and is more resistant to support women who disobey the assigned roles [52, 53]. This has been related to a major tolerance toward men who exert IPVAW, attributing to them less responsibility for the crime, while holding more victim blaming attitudes towards women [29]. Thus, in our study, the presence of more negative or passive non-helping behaviors and victim blaming attitudes among right-wing people is consistent with previous studies pointing out that victim blaming attitudes were negatively related with active bystander behaviors [22, 23]. Nonetheless, our study also found that right-wing people would confront the aggressors in greater measure than left wing people. Thus, in line with the study conducted by León et al [16], a part of this group would both intervene and hold victim blaming attitudes. Although it seems paradoxical, the greatest confrontational responses in right-wing ideology could be linked to a “protective masculinity” in the case of some men. In this regard, it should be noted that right-wing ideology has been theoretically related to such masculinity, which implies protecting vulnerable people, while their authority is recognized by victims [54, 55].

Regarding the effect that the number of bystanders could have on attitudes and behaviors, there is no variance between scenarios. Focusing on the IPVAW scenario, when participants were the only witnesses, they perceived the IPVAW scenario as more severe and considered themselves more responsible to intervene than participants who witnessed the scenario with other bystanders. However, the intention to display the different active and negative or passive non-helping behaviors did not differ on the basis of the number of bystanders, except for *do nothing because it is not my concern* which was greater in the multiple bystander scenario. Thus, our findings would be closer to those studies that found that the bystander effect takes place under certain situational conditions [56, 57], but in our case it depends on a personal condition: considering IPVAW a private matter.

Finally, it should be noted that in the IPVAW scenario as compared to the robbery situation, the number of participants stating they would actively help victims was higher than those who would not. It could be that group membership (the victim being a neighbor) is behind this fact [51].

## Study 2. Predictors of bystanders’ response intention to help IPVAW victims

### Method

#### Participants

An opportunity sample of 755 Spanish people, 595 women (78.8%) and 160 men (21.2%) with an average age of 32.90 years (*SD* = 15.26; range: 18–77) took part in this study. Nearly half of the sample had university studies (47.5%) followed by secondary studies (34.2%). Among the participants, 59.5% identified themselves as of left-wing ideology, 8.3% as right-wing ideology and 14.6% as center, and 17.6% added some other option.

#### Procedure

The procedure to recruit the sample for this exploratory study was the same as Study 1 and participants had to meet the same criteria. The recruitment period for this study started on February 24, 2022 and ended on April 24, 2022.

In this case, only the responses related to the hypothetic IPVAW scenario were used. The response rate was 63.84% (761 people completed the questionnaires from 1,192 that initiated them), a high rate compared with the average obtained by Wu, Zhao and Fils-Aime [43]. Among the respondents, only a 0.79% of the respondents (6 people) identified themselves as outside of the male/female gender binary. Due to this insufficient sample size, they were not included in this study.

#### Instruments

The same instruments were administered as in Study 1; that is, the sociodemographic data sheet, social desirability scale (variable: Social desirability) and the QIHVC (variables: Perceived severity, Victim’s Responsibility, Aggressor’s Responsibility, Bystander’s Responsibility, number of bystanders and the 8 helping behaviors). Additional questionnaires were fulfilled, as described below.

*Inventory of distorted thoughts about women and violence (IPDMV)*. The IPDMV [58]; adapted by Ferrer-Pérez et al [59] to the Spanish context measures sexist attitudes. It comprises 24 items grouped in four dimensions: Inferiority of women compared to men (IPDMV- F1, 7 items, α = 0.86); Blaming female victims of abuse (IPDMV-F2, 7 items, α = 0.62); Violence as an appropriate problem-solving strategy (IPDMV-F3, 5 items, α = 0.69); and Minimization and exoneration of the abuser (IPDMV-F4, 4 items, α = 0.53). In all scales, higher scores indicate higher levels of distorted thoughts.

*Scale on gender ideology (GI)* [60]. This scale evaluates prescriptive beliefs about the roles of women and men (gender ideology, GI) and it was also designed in a Spanish context. The reduced version of 12 items was used, which, according to its authors, has adequate psychometric properties (α > 0.70 and high correlations with similar scales). A higher score indicates a higher level of disagreement with gender equality.

#### Data analysis

In order to explore which factors are predictors of the helping behaviors, we performed a stepwise multiple regression analysis. More specifically, we explored the predictive capacity of 11 independent variables (Social Desirability, Perceived severity, Victim’s Responsibility, Aggressor’s Responsibility, Bystander’s Responsibility, number of bystanders, IPDMV-F1, IPDMV-F2, IPDMV-F3, IPDMV-F4 and GI) on each of the eight response intentions. Note that the variable number of bystanders was coded as a dummy variable (0 = only one / 1 = more than one). Given that in study 1, significant difference was found in the intention to perform most of the helping behaviors according to participants’ gender (see Table 2), we decided to carry out these analyzes separately for women and men. As a measure of effect size, we used R square, where R2 = .02 indicates a small effect, R2 = .13 a medium effect and R2 = .26 a large effect [47]. Data analysis was performed using the SPSS v. 25.

### Results

Results on the predictors of the behaviors for women can be found in Table 6 and for men in Table 7. The most powerful predictor of the intention *to reproach the victim* for their way of acting is, for both men and women, to what extent they consider the IPVAW victim responsible for the violence they have experienced. Other predictors in the case of women were the acceptance of violence as an appropriate way to resolve conflicts and, in the case of men, the existence of more than one bystander and blaming the victims.

**Table 6:** Significant predictors for women sample.

Dependent variable	Independent variables (statistically significant)	Rxy	R^2^	R^2^ change	β
Reproach the victim	Victim’s	.395	.156		+ .371
	IPDMV-F3. Violence as an Appropriate Problem-solving Strategy	.420	.177	.021	+ .146
Confront perpetrator	Bystander’s Responsibility	.307	.094		+ .320
	IPDMV-F2. Blaming Female Victims of Abuse	.321	.103	.009	+ .095
Call the police	Bystander’s Responsibility	.385	.148		+ .294
	Perceived Severity	.438	.192	.043	+ .194
	Victim’s Responsibility	.449	.202	.010	- .104
	Social Desirability	.456	.208	.006	+ .078
Help the victim	Bystander’s Responsibility	.341	.116		+ .272
	Aggressor’s Responsibility	.370	.137	.021	+ .092
	Victim’s Responsibility	.382	.146	.009	- .097
	Perceived Severity	.390	.152	.006	+ .086
Ask for help	Bystander’s Responsibility	.362	.131		+ .314
	Perceived Severity	.375	.140	.011	+ .103
	IPDMV-F3. Violence as an Appropriate Problem-solving Strategy	.384	.148	.007	- .085
Get blocked	Bystander’s Responsibility	.136	.019		- .130
	Social Desirability	.169	.029	.010	- .101
It’s not my business	Bystander’s Responsibility	.301	.090		-.257
	IPDMV-F2. Blaming Female Victims of Abuse	.341	.116	.026	+.131
	Number of bystanders [> than one]	.361	.131	.014	+.115
	IPDMV-F3. Violence as an Appropriate Problem-solving Strategy	.372	.138	.008	+.095
Nothing out for fear	IPDMV-F3. Violence as an Appropriate Problem-solving Strategy	.181	.033		+ .125
	Bystander’s Responsibility	.227	.051	.019	- .126
	Gender ideology [sexist]	.248	.061	.010	+ .108

**Table 7:** Significant predictors for men sample.

Dependent variable	Independent variables (statistically significant)	Rxy	R^2^	R^2^ change	β
Reproach the victim	Victim’s Responsibility	.394	.155		+ .367
	Number of bystanders [> than one]	.433	.187	.032	+ .187
	IPDMV-F2. Blaming Female Victims of Abuse	.457	.208	.021	+ .153
Confront perpetrator	Bystander’s Responsibility	.394	.155		+ .408
	Victim’s Responsibility	.433	.187	.032	- .180
Call the police	Bystander’s Responsibility	.437	.191		+ .302
	Perceived Severity	.512	.262	.072	+ .223
	Aggressor’s Responsibility	.536	.287	.024	+ .176
Help the victim	Bystander’s Responsibility	.483	.233		+ .376
	Aggressor’s Responsibility	.575	.331	.098	+ .331
Ask for help	Bystander’s Responsibility	.338	.114		+ .200
	Perceived Severity	.406	.165	.051	+ .213
	Victim’s Responsibility	.448	.201	.036	- .201
Get blocked	Social Desirability	.225	.051		- .198
	Bystander’s Responsibility	.273	.075	.024	- .156
It’s not my business	Bystander’s Responsibility		.181		-.368
	Victim’s Responsibility		.212	.031	+.185
Nothing out for fear	No significant predictors				

The most powerful predictor of the intention *to confront the perpetrator* of IPVAW among women and, to a greater extent, among men, was the responsibility they attribute to the bystander to act in the case of witnessing this violence. Additionally, the probability of confronting the aggressor increases among women who blame IPVAW victims but decreases among men who blame them for the violence they suffer.

With regard to the response *call the police /notify to the authorities*, the two most powerful predictors among men and women were the responsibility they attribute to the bystander to act in the event of witnessing an IPVAW situation and the perceived seriousness of this violence. Furthermore, among women, this response decreases in those cases in which the victim is blamed for the violence suffered and increases directly with social desirability. Among men, an additional predictor of this response is holding the aggressor responsible for the violence committed.

Also, in the case of the response to *help the victim*, the most powerful predictor among women and men is the responsibility they attribute to the bystander to act in case of witnessing this violence, followed by holding the aggressor responsible for the violence committed. Furthermore, among women, this response decreases in those cases in which the victim is blamed for the violence suffered and increases to the extent that IPVAW is perceived as serious.

Likewise, in the case of the response *to ask other people for help*, the most powerful predictor between women and men was the responsibility that they attribute to the bystander to act in the event of witnessing this violence, followed by the perceived severity of the IPVAW scenario. In addition, among women, this helping response decreases in those cases in which violence is accepted as an adequate way to resolve conflicts, while among men it decreases in those cases in which the victim is held responsible for the violence suffered.

With regard to *not know what to do*, *get blocked* both men and women are more likely to freeze-up in an IPVAW situation: the less responsible they feel as a bystander for intervening and the lower their susceptibility to social desirability, the more likely they are to freeze-up; however, the predictor with greater weight for women was the responsibility as a bystander and, for men, social desirability.

Likewise, both men and women are more likely to ignore the situation by *doing nothing because it’s not my business* the less responsible they feel as bystanders for intervening. In addition, the probability of this response increases in the case of women when they blame the victims, when other bystanders are present, and when they accept violence as an appropriate way to resolve conflicts. In the case of men, it is when they hold the victim responsible for the violence they suffer.

Finally, with respect to the helping behavior *doing nothing out of fear*, in the case of men, none of the variables analyzed turned out to be a significant predictor. Among women, the probability of responding this way increased to the extent that they accepted violence as an appropriate way to resolve conflicts, held a sexist ideology and felt less responsible as bystanders to intervene.

### Discussion

In Gracia’s et al [24] latest review of nearly two decades of studies on attitudes towards IPVAW in Europe, research focusing on attitudes as predictors of bystander behaviors was not found. In this study we analyzed the most frequent attitude-related variables studied in the scientific literature [24] as predictors of specific helping behaviors.

How bystanders perceived their own responsibility emerged as the strongest predictor of the intention to perform all four active helping behaviors. It was also a predictor of all negative or passive behaviors except for *reproaching the victim* and *doing nothing for fear* in the case of men. That is, in general terms, the lower the perceived responsibility of acting, the higher the chances of displaying negative or passive non-helping behaviors, while the higher the perceived responsibility of acting the higher the chances of displaying active helping behaviors. This kind of results suggests that social media campaigns should focus on boosting citizens’ personal responsibility in intervening in IPVAW contexts. Instead, previous campaigns launched worldwide have called for the spectators’ social responsibility to act, stressing the fact that IPVAW is a social issue, or trying to foresee the guilt they would feel if a tragedy were to occur (i.e. [61–64]). Indeed, as our results confirm, perceiving personal responsibility to intervene is a central key factor to activate prosocial behaviors [11, 18, 65, 66]. However, considering that previous studies point out that other variables such as victim blaming attitudes [25, 67] or in-group helping norm identification [68] can affect or predict personal perceived responsibility to act, campaigns should also focus on these issues too when calling for people to act. In fact, in our study, perceiving the victim of the IPVAW scenario responsible for the violence suffered and holding, in general terms, victim-blaming attitudes were also predictors of greater intention to *reproach the victim* or *doing nothing because it is a private matter* (for men and women). They were, as well, predictors of lesser intention *to confront* (men and women), *call the police* (women), *ask for help* (men) and *help the victim* (women). In contrast, the attribution of responsibility to aggressors was only a mild predictor of *calling the police* for men and a strongest predictor of *helping the victims* for both men and women. Our results are only partially in line with those of Wijaya et al [25], which found that the perceived responsibility of the victim and the perpetrator both accounted for the only variance in willingness to intervene in an IPVAW situation. Maybe the fact that the study presented a different scenario (IPVAW took place in a public scenario) and that willingness to intervene was captured by means of a general measure, could explain the different predictive capacity found in the perpetrator’s responsibility attribution.

It is noteworthy that although studies show that women accept and justify violence less than men [24], our study showed that, only in the case of bystander women, the acceptance of violence as a method to resolve conflicts was a predictor of *reproaching the victim*, *doing nothing because it was not of their concern* and *doing nothing for fear*. Although coherent, since people with attitudes supporting the use of violence are less likely to report it to the police (see [24, 69]), more studies need to further explore this result. Maybe the women who perceive violence as inevitable tend to respond in a negative or passive way.

Regarding the number of bystanders, this variable was not an outstanding predictor of active and negative or passive non-helping behaviors coinciding with the finds of Wijaya et al [25]. The bystander effect only played a role in the intention to *do nothing because it is not of their concern* in the case of women and *reproach the victim* in the case of men. Thus, again, it seems that attitudinal variables surround the activation of the bystander effect. Being aware of the problem and on how IPVAW traps victims (to reduce victim blaming attitudes) could help reduce the bystander effect.

Finally, the perceived severity of the IPVAW scenario was found to predict *calling the police* and *asking for help* for both women and men. Perceiving the situation as severe has been considered a pre-conditional factor to display active helping behaviors [11, 23]. According to our study, it plays a role in activating certain helping behaviors, specifically those that would be displayed in difficult situations that require the involvement of more people or of the authorities. It was also found that the perceived severity was a predictor of *helping the victim* only for women. Again, self-identification processes could play a role, that is, women who witness an IPVAW scenario could identify themselves in greater measure with the victims than men, which could increase the chances of helping the victims, especially when the violence is perceived as severe. It could also be that women perceive that they have the ability to directly help the victims, since they are socialized in caring behaviors [70]. It should be noted that having abilities is also a conditional factor to actively help victims, according to Latané and Darley’s model [11].

## General conclusions

A considerable percentage of people are witnesses of IPVAW [4, 50]. However, in Spain, the amount of research is limited [50]. Advancing in this area is a priority, since promoting people’s willingness to intervene is a strategy that can help advance towards the eradication of IPVAW [7, 8]. This study provides further knowledge on how the most relevant and potentially relevant factors contemplated in the bystander literature in IPVAW contexts relate to specific active and negative or passive non-helping behaviors according to the bystander’s gender, the bystander effect and political ideology, as well as on the predictors of such behaviors. In line with previous studies [i.e. 24, 50], women tend to help more, especially in a non-confrontational way, perceive IPVAW as more severe, perceive themselves more responsible to act and hold fewer victim blaming attitudes. Further studies are needed to discern whether the differences found are due to, for instance, group membership or to a major awareness on the issue. With regard to the bystander effect, it only takes place when people do not intervene because they consider IPVAW a private matter, thus when negative attitudes are present. Therefore, this type of attitude still needs to be changed. Maybe campaigns focused on providing arguments on why IPVAW is a social issue could help (i.e. alluding to economic costs). An individual’s political opinion has also shown to affect the assessment and, to a lesser extent, the intention to help an IPVAW victim. Perhaps campaigns could make people aware of this and promote differentiating a crime from how people would like the social order to be. However, no differences have been found on the assessment and intention to help a victim when comparing IPVAW with a common form of violence. This result is very positive since our participants were highly willing to help victims in both cases, although considering the intention-behavior gap (see [42]) further studies are needed to know whether intentions turn into actions in both situations. When analyzing the interaction between the type of violence and other variables, the present results tend to confirm previous: in the IPVAW scenario, women tend to assess and respond in a positive way; left-wing people perceive the situation as more severe and perceive a greater responsibility to act; and the bystander effect does not occur.

Regarding the predictors of the helping behaviors, perceived personal responsibility is a key variable on which campaigns should focus although other predictors such as holding victim blaming attitudes or the perceived severity of the situation are likewise relevant. Victim blaming attitudes hinder helping actively, which is why it is also important to target them in campaigns. Also, for a person to perceive personal responsibility to act, he/she previously needs to perceive the situation as severe [11, 23].

These studies also present several limitations. In the first place, regarding the sampling method used (opportunity sample) and the skewed distribution of sociodemographic variables, it can affect the representativeness of the sample and the external validity of results. In fact, there is a large disparity between the number of women and men that participate in the studies, most of the participants have university studies and hold left-wing ideas. This sample composition means that the results obtained cannot be generalized to the population, especially to groups with a lower level of education. In the second place, participants had to complete several questionnaires which could affect responses. Nonetheless, they could answer the questionnaire at different moments. Additionally, some scales used in the study exhibit a low internal consistency. However, reliability values around .50 can be considered acceptable in basic research studies [71, 72]. And, finally, the format (online) has several disadvantages, such as not being able to respond to doubts, although it allows access to a greater number of participants with fewer costs.

We would like to highlight two main strengths. In the first place, based on the reviewed literature, these are the first studies in Spain that provide knowledge on how the variables of greatest (and potential) interest studied in the bystander literature can affect different helping behaviors, as well as which are the predictors of such helping behaviors. Studies have analyzed a limited number of variables related to bystander behaviors with contrary results [17, 19–22, 36], and only few have focused on identifying predictors of bystander behaviors in IPVAW scenarios [25, 39, 41, 49]. In the second place, the findings allow advancing in the design of evidence-based campaigns. Public media needs to engage citizens in helping end intimate partner violence against women (IPVAW) [73].
